# Multidisciplinary Intervention in Patients with Musculoskeletal Pain: a Randomized Clinical Trial

**DOI:** 10.1007/s12529-015-9486-y

**Published:** 2015-04-29

**Authors:** Randi Brendbekken, Anette Harris, Holger Ursin, Hege R. Eriksen, Tone Tangen

**Affiliations:** Department of Physical Medicine and Rehabilitation, Innlandet Hospital Trust, Ottestad, Norway; Department of Health Promotion and Development, University of Bergen, Bergen, Norway; Uni Research Health, Bergen, Norway; Department of Clinical Medicine, Haukeland University Hospital, University of Bergen, Bergen, Norway

**Keywords:** Randomized clinical trial, Chronic musculoskeletal pain, Multidisciplinary treatment, Patient education tool

## Abstract

**Background:**

Musculoskeletal pain is associated with comorbidity, extensive use of health services, long-term disability and reduced quality of life. The scientific literature on effects of treatment for musculoskeletal pain is inconclusive.

**Purpose:**

The purpose of this study is to compare a multidisciplinary intervention (MI), including use of the novel Interdisciplinary Structured Interview with a Visual Educational Tool (ISIVET), with a brief intervention (BI), on effects on mental and physical symptoms, functioning ability, use of health services and coping in patients sick-listed due to musculoskeletal pain.

**Method:**

Two hundred eighty-four adults aged 18–60, referred to a specialist clinic in physical rehabilitation, were randomized to MI or BI. Patients received a medical examination at baseline and completed a comprehensive questionnaire at baseline, 3 months and 12 months.

**Results:**

Both groups reported improvements in mental and physical symptoms, including pain, and improved functioning ability at 3 and 12 months, but the MI group improved faster than the BI group except from reports of pain, which had a similar course. Significant interactions between group and time were found on mental symptoms (anxiety (*p* < 0.05), depression (*p* < 0.01), somatization (*p* < 0.01)) and functioning ability (*p* < 0.01) due to stronger effects in the MI group at 3 months. At 3 and 12 months, the MI group reported significantly less use of health services (general practitioner (*p* < 0.05)). At 12 months, the MI group reported better self-evaluated capability of coping with complaints (*p* < 0.001) and they took better care of their own health (*p* < 0.001), compared to the BI group.

**Conclusion:**

The results indicate that the MI may represent an important supplement in the treatment of musculoskeletal pain.

## Introduction

Musculoskeletal pain conditions such as fibromyalgia and low back pain are, in the majority of cases, unspecific and composite [1]. Although non-malignant, they represent substantial suffering and economic loss for the individual itself and for the society due to frequent contacts with the health care system, absence from work and reduced quality of life [2–6]. In Norway, musculoskeletal diagnoses represent about 45 % of the long-term sick leave [7]. Most of the patients have other subjective health complaints as well, where pathological findings are absent or substantially less than expected, compared to the reported intensity of the complaints [8]. There is general consensus in the literature that these conditions are multicausal [9] and comorbid [8, 10, 11]. Psychological and social factors, as well as somatic pathology, influence chronicity and disability [12]. This indicates that the optimal treatment should focus on several aspects of the patient’s life [1, 12, 13]. Improved incorporation of patient preferences into treatment recommendations can improve adherence to treatment and thereby improve the clinical outcomes [14]. Multidisciplinary treatment is a well-accepted and well-documented method to treat chronic pain [13, 15, 16], and education combined with physical exercise produces some positive effects in long-term follow-up for fibromyalgia and musculoskeletal pain [13]. The European guidelines for low back pain state that the optimal content of multidisciplinary programs requires further research, but behavioural treatment and stress management are important components of these programs [1]. There is, however, a lack of systematic content or description of such, in many of these programs.

The aim of this study was to compare the effects of a multidisciplinary intervention (MI) and a brief intervention (BI), on mental and physical health complaints, functioning ability and coping in patients on long-term sick leave due to musculoskeletal complaints. The study is part of a randomized clinical trial (trial reg. nr. NCT01346423) where return to work was the main outcome.

## Material and Methods

**Fig. 1:**
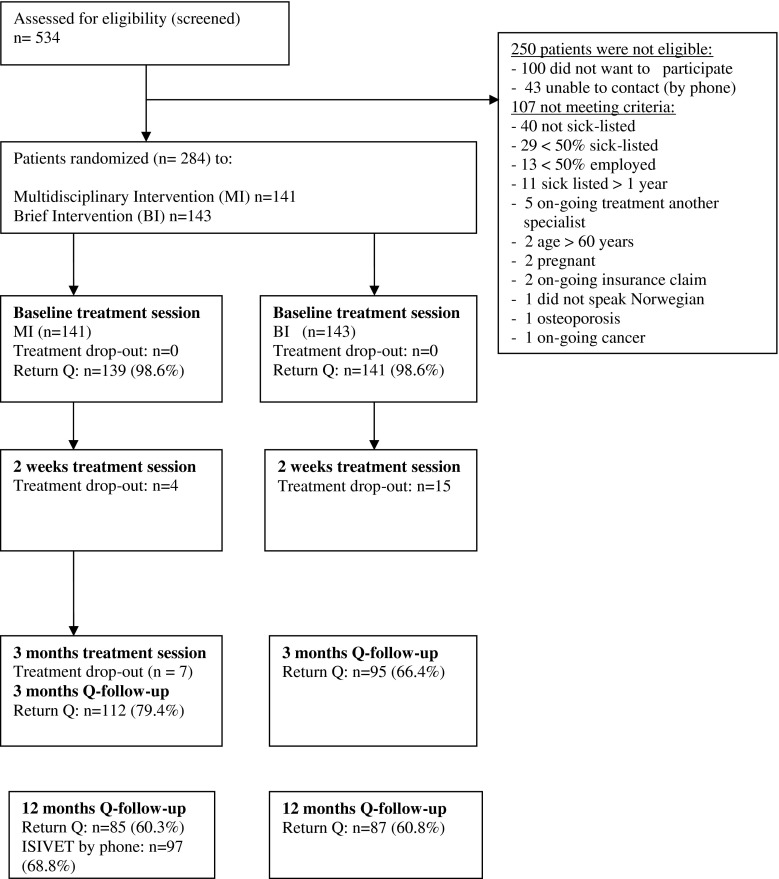
Flowchart of participation, in treatment sessions and questionnaires (Q) follow-up

Five hundred thirty-four patients with musculoskeletal pain referred to a specialist outpatient clinic, at the Department of Physical Medicine and Rehabilitation, Innlandet Hospital Trust, Norway, between 2011 and 2013, were considered for participation in the trial. Patients were referred from general practitioners (GPs) in 48 municipalities in two different counties in the south-eastern part of Norway. The inclusion criteria were as follows: age between 20 and 60 years, at least 50 % sick leave due to musculoskeletal pain for less than 12 months and at least 50 % employed. The exclusion criteria were as follows: pregnancy, current cancer, osteoporosis, recent physical trauma/injury, serious mental illness, rheumatic inflammatory diseases, not capable of understanding and speaking Norwegian or being involved in an on-going health insurance claim. Of the 534 patients, 250 were either not eligible or excluded for different reasons. Two hundred eighty-four (mean age 41.3 years, 54 % females) patients were included in the study and randomized to either MI (*n* = 141) or BI (*n* = 143) (Fig. 1). The patients included were referred from 136 different GPs who referred between one and eight patients each.

### Interventions

The two interventions were given by different teams and at two different outpatient clinics.

#### The MI with the ISIVET

##### Baseline Treatment Session

A social worker, a physician and a physiotherapist performed the MI. Initially, they consulted the patient successively. Each consultation was two-parted: First, an interview and, eventually, a physical examination and, secondly, the use of ISIVET.

In the first part, the social worker interviewed the patient on her/his social situation (family life, social life, education, economics) and work situation, while the physician did a comprehensive interview covering past and present physical and mental health for the patient and his/her family. The physician also elaborated on coping and fear avoidance in relation to the pain problems, in addition to a physical examination, concluding with a diagnosis according to ICD-10. The physiotherapist assessed the musculoskeletal problems of the patient through interview and a physical examination.

**Fig. 2:**
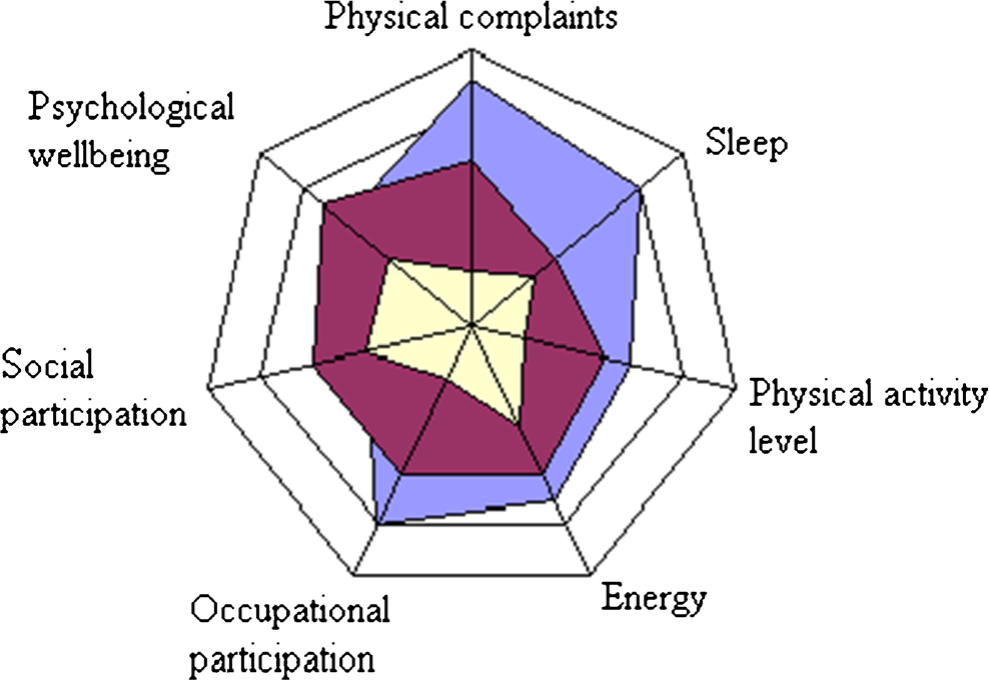
Example of figure “*quality of life*” filled in three times

During the second part of each consultation, the therapists used the ISIVET. The method is developed by the first author (RB) and consists of two figures, a manual, a table for filling out a rehabilitation plan and a list where possible rehabilitation initiatives are categorized. Each figure is a star plot with seven axes representing different variables (Fig. 2). Each axis has the range from 1 (centrally) to 10 (peripherally). The patient scored her/himself with assistance from the therapist and guiding from the manual, on each variable on this numeric scale, where “1” indicates a maximum negative situation whereas “10” indicates an optimally positive situation. The manual gives illustrating examples of the situation at different levels. Patient and therapist read the manual together, and through discussion, they identified the right score for each variable and marked it on the actual axis in a paper version of the figure. When all scores were completed, a line was drawn between the seven scores giving an area in each of the two figures. The area under the lines was coloured for better visualization for the patient as well as for the therapists. Problem areas or challenges were demonstrated as lack of colour, while existing resources stood out as coloured area.

The first figure “working conditions” included the following variables: work-related stress, satisfaction with job tasks, workload, collegial relationships, leadership, degree of challenges at work and occupational participation. It was filled out during the consultation with the social worker. The second figure “quality of life” included the following variables: physical complaints, psychological well-being, sleep, energy, physical activity, social participation and occupational participation and was filled out during the consultation with the physician and the physiotherapist.

When the sessions with the three therapists were completed, the whole team met briefly, sharing their findings and general impression of the patient and her/his situation at work and at home. Possible barriers to work participation, maintaining factors to the on-going pain problems and, eventually, other important issues were highlighted. The patient then joined the meeting to a final discussion with the team on her/his situation, health problems and work situation. The two figures from the ISIVET were central when discussing possible fields of actions. The patient decided ways to go forward, and agreement on actions was written down in a table and categorized according to the standardized protocol. These action items comprised the patient’s rehabilitation plan and were typically related to cognitive assessment of health, as fear avoidance and catastrophic thinking, lifestyle and, if relevant, family and work matters. Actions could also involve physical exercise or increased daily physical activity. When leaving the clinic, the patient received a paper copy of the figures with the coloured areas and the rehabilitation plan listed as points to be followed. A copy of the complete medical record was sent both to the GP and the patient. The baseline assessment lasted about 3.5 h.

- *After 2 weeks*, the patient and the physiotherapist met for about 1 h to evaluate the rehabilitation plan and work through the ISIVET once more. New scores and new areas were marked in the figures made at baseline, and areas were coloured with a new colour (Fig. 2). Visualization of delta areas in the star plot was matter of attention and reflection. Previous advice and actions were highlighted according to this, and adjustments in the rehabilitation plan were eventually made.
- *After 3 months*, the patient met with the whole team for about 1 h to sum up the situation and evaluate the interventions so far. The ISIVET was worked through, and new areas on the figures were coloured with a third colour. Eventually, they adjusted the rehabilitation plan.
- At *12 months follow-up*, the physiotherapist contacted the patient by phone to score the two figures in the ISIVET a last time. This was a brief contact that lasted about 15 min.

To ensure adherence to the protocol and equal practice of the method, the MI team had regular meetings for supervision and discussions. Four physicians, all specializing in physical medicine and rehabilitation, two social workers and four physiotherapists did the treatment. The team members were the same during the treatment course of one patient. Audiotaping was not used.

#### The BI

BI is a standardized intervention based on the studies by Indahl [17] and Molde Hagen [18], and details on the method are described in the pioneer work of Indahl [10, 17].

BI comprised of two sessions. The baseline session lasted about 2.5 h and included separate consultations with the physician and the physiotherapist. After 2 weeks, the patients had a follow-up session with the physiotherapist for about 1 h.

The basic principle of the BI is the non-injury model, emphasizing the lack of any objective signs of injury [17, 19] and the non-directive communication [20, 21]. BI has proven more effective on return to work (RTW), health complaints and functional ability, than usual care both for chronic low back pain and non-specific muscular pain conditions [18, 20]. The goal is to reduce fear and concern through a thorough medical examination with explanations of each step and education about a physiological model on musculoskeletal pain. Any somatic findings are explained. The patient is informed about the good prognosis and the importance of staying active.

A physician who was specialist in physical medicine and rehabilitation and a physiotherapist did the BI. Both were experienced in the method. Therapist treatment manuals were written for the intervention, based on current guidelines [1] and on the manuals used by Indahl and Molde Hagen [10, 18]. Audiotaping was not used. The physician had been videotaped giving BI in another trial [22]. A copy of the medical record was sent both to the GP and the patient.

### Randomization and Blinding

The randomization was concealed, and patients were randomized to either MI or BI, according to a computer-generated randomization list set-up by a statistician at Uni Research Health (URH). URH received information on ID number, gender and age, and a research assistant, not involved in the treatment, contacted URH and was informed on which treatment that the patient should receive. For practical reasons, there was no blinding to treatment of therapists or participants.

### Dropout from Randomized Treatment

Patients who dropped out of treatment were asked if they were willing to continue filling out questionnaires and return them by mail.

### Questionnaires

The patients received the questionnaires by mail and filled them out at baseline and at 3 and 12 months follow-up. The following questionnaires were applied:

- *The Hospital Anxiety and Depression Scale* (*HADS*) covered symptoms of anxiety and depression [23]. HADS consists of 14 items, of which seven measures anxiety and seven depressive symptoms. Scores are made on a four-point Likert scale ranging from 0 (“not at all”) to 3 (“very often”) on symptoms experienced during the last week, providing 21 as a maximum sum score for each subscale. A cut-off score of eight and above is used as an indication of possible, anxiety or depressive disorder.
- *The Hopkins Symptom Checklist-25* (*HSCL-25*) measures psychological distress [24]. The instrument consists of 25 questions recording the presence and intensity of the most common symptoms of anxiety, depression and somatization. Severity is scored on a four-point Likert scale from 1 (not at all) to 4 (“very much/to a severe degree”), and a mean score <1.75 is within normal range, while a score ≥1.75 indicates psychological distress in the need of treatment.
- *The Norwegian Function Assessment Scale* (*Norfunk*) measures four aspects of physical function and three aspects of psychological function during the last week by 41 questions [25]. Physical function is related to the patient’s ability to walk/stand, hold/pick, lift/carry and sit. Psychological function covers the ability to be attentive, communicate, work in team, handle responsibility, handle challenges of daily life, deal with criticism, cope with anger, communicate with others and to look/listen. The answers are scored on a four-point Likert scale from 0 (“no problems”) to 3 (“not able to do the activity”).
- *Subjective Health Complaints* (*SHC*) *inventory* is a reliable instrument measuring somatic and psychological complaints experienced during the last month [26]. It contains 29 items covering the most frequent subjective health complaints from different parts of the body. Severity is scored on a four-point Likert scale from 0 (not at all) to 3 (“seriously”). The instrument has five subscales: musculoskeletal complaints, gastrointestinal problems, pseudoneurological problems, flu and allergy symptoms in addition to a total score (SHC total).
- *Pain* was measured with a Numeric Rating Scale (NRS). The patients were asked about mean pain in the back, the neck, the foot and during activity, at rest and at night for the last 14 days. The severity of pain was scored from 0 (“no pain”) to 10 (“worst possible pain”).

At 3 and 12 months, the patients were asked if they had been treated by GP, chiropractor or physiotherapist or had received other treatment during the last 3 months and, if so, for how many sessions.

At 12 months, the patients were also asked about changes, compared to 1 year ago, in complaints, general health, coping with health complaints, ability to take care of their own health and physical fitness. They were also asked about satisfaction with the treatment. Answers were scored on a five-point Likert scale from 1 (“much better”) to 5 (“much worse”) except from patient satisfaction with treatment which was assessed on a seven-point Likert scale from 1 (“very satisfied”) to 7 (“very dissatisfied”).

### Statistics

A mixed between–within-subjects analyses of variance with one between-group factor (MI vs. BI) and one within-subjects/repeated measures factor (baseline, 3 months, 12 months) were conducted to assess the effect of the two interventions on participant scores on depression, anxiety, somatization (HADS and HSCL), functional ability (Norfunk) and health complaints (SHC). The interaction effects (time × group) were calculated, and when significant, such interaction effects indicate different time courses for the two interventions. Interaction effects were followed up by *t* tests for paired samples within each group. Cohen’s *d* was calculated between baseline and 3 months follow-up and baseline and 12 months follow-up using an online calculator (http://easycalculation.com/statistics/effect-size.php) based on this formula: *d* = *M*_1_ − *M*_2_ / (√(SD_1_^2^ + SD_2_^2^) / 2). Differences for outcomes between the two interventions in scores on pain measured by NRS were analyzed by Mann–Whitney *U* test for independent samples at 3 and 12 months. Differences for outcomes in use of health services, patient-evaluated health changes, coping and satisfaction with treatment at 12 months were assessed with *x*^2^ statistics or Fisher’s exact test. Sample size calculations were in accordance with RTW expectations, which are part of this RCT but described in another paper, and based on data from Hagen et al. [18]. The calculation was based on a power of 80 % and a significant level of 5 % giving an *N* for this study of 300.

### Ethical Considerations

The study followed the Helsinki declaration and was approved by the Norwegian Regional Ethics Committee in south-eastern Norway [27] and by the Norwegian Social Science Data Services [28]. Participants gave their informed consent by signing the declaration of voluntarily participation before entering in the study.

## Results

### Demographic and Baseline Data

The study population comprised 284 individuals (mean age 41.3 years, 53.9 % women). Two hundred seventeen (76.4 %) of the patients were married or cohabitant, 195 patients (68.7 %) reported education limited to primary school (≤12 years), 56 patients (19.7 %) had no children, and 238 patients (83.8 %) reported 80 % employment or more. Mean duration of sick leave during the 8-month period before entry to the study was 143 days (SD = 56.6) in the MI group and 150 days (SD = 62.9) in the BI group. The dominant diagnoses in accordance to ICPC-2 [29] were as follows: low back pain L02/L03/L84/L86 (39.5 %), neck pain L01/L83 (12.1 %), widespread pain/fibromyalgia L18 (10.7 %) and shoulder pain L08/L92 (7.8 %). The whole study population constituted 51 different diagnoses, the L group representing 84.2 %. There were no differences in pain diagnoses between the groups at baseline.

### Lost to Follow-Up

The dropout of treatment was low in both groups (Fig. 1). Return of questionnaires dropped to 60.3 % in the MI group and 60.8 % in the BI group at 12 months (Fig. 1). There were no significant differences in baseline scores between returners and non-returners of questionnaires, except from the score on HSCL depression where the non-returners scored significantly lower (mean = 1.47, SD = 0.46) compared to the returners (mean = 1.60, SD = 0.55), giving a *p* value <0.05 of the difference.

### Changes in Anxiety, Depression and Somatization

Anxiety and depression measured with HADS and somatization and depression measured with HSCL showed a significant interaction between group and time, indicating that the BI group and the MI group differed significantly on these subscales (Table 1). By 3 months, the MI group reported improvements on anxiety, depression and somatization (all *p* values <0.01) measured with HADS and HSCL, while the BI group reported a significant worsening on HADS anxiety (*p* < 0.01) and a smaller improvement on anxiety, depression and somatization measured with HSCL (all *p* values < 0.05) compared to the MI group. However, at 12 months, the groups were similar, with both groups reporting significantly improvements on all subscales.

**Table 1:** Effects of multidisciplinary intervention (MI) and brief intervention (BI) on anxiety, depression and somatization measured by the Hospital Anxiety and Depression Scale (HADS) and Hopkins Symptom Checklist (HSCL)

	MI		BI		Interaction effect^c^ (time × group)	
	Mean (SD)^a^	*d* ^b^	Mean (SD)^a^	*d* ^b^	*F* value	*p* value
HADS anxiety						
Baseline^d^	5.59 (3.29)		5.51 (3.70)			
3 months^e^	4.82 (3.34)**	0.27	5.74 (4.12)**	−0.02		
12 months^f^	4.53 (4.25)**	0.24	4.79 (4.08)**	0.28	*3.79*	*0.02*
HADS depression						
Baseline^d^	4.58 (3.42)		4.50 (3.55)			
3 months^e^	3.83 (3.35)**	0.32	4.86 (4.11)	−0.06		
12 months^f^	3.71 (3.85)**	0.21	3.99 (3.65)*	0.23	*10.89*	<*0.00*
HSCL somatization						
Baseline^d^	2.01 (0.54)		1.95 (0.58)			
3 months^e^	1.74 (0.49)**	0.63	1.87 (0.70)*	0.15		
12 months^f^	1.69 (0.57)**	0.61	1.73 (0.67)**	0.40	*8.01*	<*0.00*
HSCL anxiety						
Baseline^d^	1.47 (0.41)		1.45 (0.40)			
3 months^e^	1.35 (0.34)**	0.38	1.42 (0.43)*	0.17		
12 months^f^	1.32 (0.39)**	0.40	1.33 (0.44)**	0.39	2.17	0.12
HSCL depression						
Baseline^d^	1.54 (0.48)		1.55 (0.56)			
3 months^e^	1.35 (0.38)**	0.50	1.50 (0.58)*	0.19		
12 months^f^	1.39 (0.49)**	0.36	1.40 (0.59)**	0.38	*4.14*	*0.02*

*p < 0.05 and **p < 0.01 based on paired samples *t* test within each group compared with baseline assessment

^a^Paired *t* test, comparing baseline and 3 months, and baseline and 12 months. Separate tests for the BI group and the MI group

^b^Cohen’s *d* for paired values. A negative Cohen’s *d* indicates a worsened score compared to baseline. Small effect *d* = 0.2, medium effect *d* = 0.5, large effect *d* = 0.8

^c^A mixed between-within-subjects analyses of variance comparing the effect of the BI and the MI intervention (Wilks’ lambda), *F* value and interaction effects. *P*-values < 0.05 indicate significant different time courses for the two interventions

^d^Baseline MI: *n* = 139 (98.6 %). BI: *n* = 141(98.6 %)

^e^3 months: MI: *n* = 112 (79.4 %). BI: *n* = 95 (66.4 %)

^f^12 months: MI: *n* = 85 (60.3 %). BI: *n* = 87 (60.8 %)

### Changes in Functional Ability

Functional ability measured with Norfunk showed a significant interaction between group and time, indicating that the BI group and the MI group had a significantly different time course on the functional ability (Table 2). The MI group had significant improvements from baseline to 3 months on six of seven subscales and on the total score (all *p* values <0.01), while the BI group had significant, but weaker improvements on two subscales (*p* < 0.05). By 3 months, the Cohen’s *d* was larger on all items of the Norfunk in the MI group compared to the BI group, which had negative value on three subscales, indicating deterioration. By 12 months, both groups had significant improvements from baseline, but with no significant differences between the groups.

**Table 2:** Effects of multidisciplinary intervention (MI) and brief intervention (BI) on different aspects of functional ability (Norfunk)

	MI		BI		Interaction effect^c^ (time × group)	
	Mean (SD)^a^	*d* ^b^	Mean (SD)^a^	*d* ^b^	*F* value	*p* value
Norfunk all items						
Baseline^d^	1.44 (0.28)		1.44 (0.30)			
3 months^e^	1.33 (0.29)**	0.43	1.40 (0.33)	0.10		
12 months^f^	1.32 (0.34)**	0.38	1.30 (0.29)**	0.51	*5.52*	*0.01*
Coping, handle responsibility, attention, concentration, work, tolerate stress						
Baseline^d^	1.44 (0.41)		1.42 (0.44)			
3 months^e^	1.31 (0.38)**	0.36	1.48 (0.55)	−0.10		
12 months^f^	1.36 (0.44)*	0.24	1.31 (0.38)*	0.27	*5.80*	*0.01*
Ability to hold, to pick, to write, to drive, to cook, to dress/undress						
Baseline^d^	1.37 (0.33)		1.36 (0.33)			
3 months^e^	1.27 (0.34)**	0.33	1.32 (0.36)	0.08		
12 months^f^	1.25 (0.35)*	0.34	1.21 (0.30)**	0.48	*3.44*	*0.04*
Ability to stand, to walk flat, to walk stairs, to shop						
Baseline^d^	1.55 (0.52)		1.58 (0.50)			
3 months^e^	1.45 (0.47)**	0.29	1.47 (0.45)	0.19		
12 months^f^	1.38 (0.48)**	0.35	1.39 (0.48)**	0.43	1.17	0.31
Ability to lift, to carry, to laundry, to housekeep						
Baseline^d^	1.73 (0.51)		1.71 (0.50)			
3 months^e^	1.53 (0.51)**	0.34	1.58 (0.50)*	0.24		
12 months^f^	1.46 (0.47)**	0.50	1.46 (0.42)**	0.63	0.99	0.37
Ability to sit, to be a passenger in car/bus/train						
Baseline^d^	1.41 (0.55)		1.42 (0.55)			
3 months^e^	1.22 (0.41)**	0.39	1.28 (0.47)*	0.21		
12 months^f^	1.22 (0.41)**	0.36	1.21.(0.37)**	0.45	1.28	0.28
Ability to communicate verbally, written and by phone, to cooperate, to perceive messengers						
Baseline^d^	1.26 (0.35)		1.26 (0.35)			
3 months^e^	1.24 (0.34)	0.08	1.32 (0.41)	−0.12		
12 months^f^	1.29 (0.40)	−0.06	1.28 (0.37)	0.00	0.76	0.47
Ability to watch TV, listen to radio						
Baseline^d^	1.07 (0.19)		1.08 (0.24)			
3 months^e^	1.06 (0.20)	0.07	1.12 (0.29)	−0.12		
12 months^f^	1.09 (0.27)	−0.18	1.07 (0.24)	0.02	*3.77*	*0.03*

*p < 0.05 and **p < 0.01 based on paired samples *t* test within each group compared with baseline assessment

^a^Paired *t* test, comparing baseline and 3 months, and baseline and 12 months. Separate tests for the BI group and the MI group

^b^Cohen’s *d* for paired values. A negative Cohen’s *d* indicates a worsened score compared to baseline. Small effect *d* = 0.2, medium effect *d* = 0.5, large effect *d* = 0.8

^c^A mixed between-within-subjects analyses of variance comparing the effect of the BI and the MI intervention (Wilks’ lambda), *F* value and interaction effects. *P*-values < 0.05 indicate significant different time courses for the two interventions

^d^Baseline MI: *n* = 139 (98.6 %). BI: *n* = 141(98.6 %)

^e^3 months: MI: *n* = 112 (79.4 %). BI: *n* = 95 (66.4 %)

^f^12 months: MI: *n* = 85 (60.3 %). BI: *n* = 87 (60.8 %)

### Changes in SHC

There were no significant interactions between group and time for any of the SHC subscales (Table 3). This indicates that the two interventions did not affect SHC differently. The Cohen’s *d* was larger on all items by 3 months in the MI group compared to the BI group, and the changes from baseline to 3 months were, overall, larger in the MI group by 3 months. By 12 months, the effect sizes were similar for the groups, due to improvements in the BI group from 3 to 12 months, leaving the two groups similar.

**Table 3:** Effects of multidisciplinary intervention (MI) and brief intervention (BI) on subjective health complaints (SHC)

	MI		BI		Interaction effect^c^ (time × group)	
	Mean (SD)^a^	*d* ^b^	Mean (SD)^a^	*d* ^b^	*F* value	*p* value
SHC total						
Baseline^d^	20.13 (9.38)		18.42 (9.39)			
3 months^e^	16.12 (8.97)**	0.48	17.34 (10.51)*	0.16		
12 months^f^	15.71(10.22)**	0.42	15.25(10.44)**	0.42	2.20	0.11
SHC musculoskeletal complaints						
Baseline^d^	10.62 (4.24)		10.07 (4.36)			
3 months^e^	8.78 (4.37)**	0.47	8.83 (4.62)**	0.30		
12 months^f^	8.22 (4.73)**	0.50	7.89 (4.79)**	0.57	1.64	0.20
SHC pseudoneurological symptoms						
Baseline^d^	4.96 (3.20)		4.79 (3.59)			
3 months^e^	3.79 (3.11)**	0.43	4.56 (3.69)	0.11		
12 months^f^	3.61 (3.57)**	0.39	3.95 (3.55)**	0.33	1.40	0.25
SHC gastrointestinal symptoms						
Baseline^d^	2.67 (2.91)		1.97 (2.53)			
3 months^e^	2.13 (2.40)*	0.24	2.16 (2.71)	0.02		
12 months^f^	2.29 (2.72)	0.15	1.94 (3.27)	0.11	0.29	0.75
SHC allergy symptoms						
Baseline^d^	1.13 (1.81)		0.91 (1.43)			
3 months^e^	0.82 (1.35)*	0.20	0.89 (1.35)	−0.03		
12 months^f^	0.74 (1.16)*	0.23	0.62 (1.08)*	0.21	2.21	0.11

*p < 0.05 and **p < 0.01 based on paired samples *t* test within each group compared with baseline assessment

^a^Paired *t* test, comparing baseline and 3 months, and baseline and 12 months. Separate tests for the BI group and the MI group

^b^Cohen’s *d* for paired values. A negative Cohen’s *d* indicates a worsened score compared to baseline. Small effect *d* = 0.2, medium effect *d* = 0.5, large effect *d* = 0.8

^c^A mixed between-within-subjects analyses of variance comparing the effect of the BI and the MI intervention (Wilks’ lambda), *F* value and interaction effects. *P*-values < 0.05 indicate significant different time courses for the two interventions

^d^Baseline MI: *n* = 139 (98.6 %). BI: *n* = 141(98.6 %)

^e^3 months: MI: *n* = 112 (79.4 %). BI: *n* = 95 (66.4 %)

^f^12 months: MI: *n* = 85 (60.3 %). BI: *n* = 87 (60.8 %)

### Changes in Pain

Pain by activity (MI group = 6.62 (1.93), BI group = 6.26 (2.11)) and back pain (MI group = 5.97 (2.28), BI group = 5.69 (2.44)) was the main pain problem in both groups at baseline. Both groups had reduction in their average pain levels during the follow-up, but there were no significant differences between the groups at 3 or 12 months on pain by activity or back pain (results not shown).

### Use of Health Services by 3 and 12 Months

By 3 and 12 months, the MI group had consulted their GP significantly less than the MI group (*p* < 0.05): By 3 months 19.4 % in the MI group and 31.8 % in the BI group had received treatment by their GP during the last 3 months, with about equal mean number of treatment sessions: MI = 3.0 and BI = 2.8. By 12 months, the corresponding numbers were 11.8 and 18.5 %, mean MI = 2.5 and BI = 2.3 (*p* < 0.05). There were no significant differences between the groups in consulting other therapists at 3 or 12 months follow-up.

### Changes in Health Complaints/Symptoms, Coping and Satisfaction with Treatment

By 12 months, there were no significant differences between the groups in self-evaluated changes in complaints (*x*^2^ (1, *n* = 171) = 3.4); 85 individuals (96.5 %) in the MI group and 86 (98.8 %) in the BI group reported that they still had musculoskeletal complaints. By 12 months, the MI group reported significantly better ability to cope with problems (*x*^2^ (1, *n* = 168) = 22.5, *p* < 0.001), better ability to take care of their own health (*x*^2^ (1, *n* = 165) = 17.3, *p* < 0.001) and better physical fitness (*x*^2^ (1, *n* = 165) = 15.1, *p* < 0.01) compared to the BI group. The MI group also reported significantly higher satisfaction with the treatment (*x*^2^ (1, *n* = 170) = 41.8, *p* < 0.001).

## Discussion

Comparing the effects of a multidisciplinary intervention (MI), including use of the novel Interdisciplinary Structured Interview with a Visual Educational Tool (ISIVET), with a brief intervention (BI) on patients sick-listed due to musculoskeletal pain, revealed no significant differences between groups on mental and physical symptoms and functional ability at 12 months follow-up. However, patients in the MI group had significantly better effect on anxiety, depression, somatization and functional ability at 3 months follow-up, compared to the BI group, and at 12 months follow-up, the MI group reported better ability to cope with their problems, higher ability to take care of their own health and better physical fitness in spite of the same level of pain, and they consulted their GP less than patients in the BI group both at 3 and 12 months.

In Norway, treatment of musculoskeletal pain is primarily done by the patients’ GP. Chronic and more complex cases are eventually referred to specialist health care [13, 30, 31]. It is reasonable to assume that our study population consists of chronic and more complex cases, as they had been sick-listed for, on average, 147 days and were referred by their GPs to specialist health care. The GPs had no prior knowledge that their patients might be enrolled into a clinical study.

Typically, episodes of acute musculoskeletal pain including LBP recover quickly, but patients who do not recover tend to have more complex disorders where social factors, work conditions, psychological and somatic factors play together in perpetuating the condition [5, 32–34]. Clinical psychosocial factors predict long-term incapacity of musculoskeletal disorders [35], and multidisciplinary treatments including a psychosocial approach have been proven effective for complex illnesses [15, 36] and are well accepted in treatment of chronic pain [13, 15, 16]. The MI patients received more extensive, multidisciplinary treatment, compared to the BI patients. This may explain why the MI was more effective than the BI at 3 months follow-up, on anxiety, depression, somatization and functional ability. The baseline mean scores on anxiety and depression were low for both groups. A tendency to somatization among these patients where they express stress in somatic symptoms rather than psychologically might indicate that changes in even low scores could be of clinical importance.

Improved communication between patient and health professionals can influence health outcomes and coping in a positive way [37–39]. ISIVET is constructed to improve communication, patient involvement, mutual understanding and enhancing of the therapeutic alliance. Filling in the ISIVET figures with the therapist may represent a communication where the patient experiences that her/his opinion and experiences are respected and made relevant, leading to a mutual insight and understanding of the situation between patient and therapist. In BI, the communication was a more traditional doctor–patient relationship where the patient was given information and advices about physical activity and exercises to improve their muscle pain.

Earlier trials have shown that patients who are engaged in decision making are more motivated for changes in lifestyle and their clinical outcomes are better [38, 40]. In shared decision making (SDM), the patient’s autonomy is strengthened and the relationship with the therapist and the patient is improved [41]. The application of ISIVET in assessment of health complaints can facilitate patient empowerment and SDM. This may lead to improvements of patient satisfaction, adherence to treatment and better health outcomes [42].

Educational tools can influence the patient’s expectations and outcomes in a beneficial way compared to traditional health information [43]. When combining education and physical exercise, there are some positive long-term effects for fibromyalgia and musculoskeletal pain [13]. Application of ISIVET where a visualization of the patient’s situation was established as coloured areas in the ISIVET figures may facilitate the patient’s insight and understanding of the complexity of the situation. This might improve the adherence to the rehabilitation plan. At 12 months, the MI group reported better ability to handle health problems and better physical fitness and they had less use of health care services in spite of fairly the same levels of pain and health complaints as the BI group.

### Limitations and Strengths of the Study

The dropout of treatment was low, but the return of questionnaires at 12 months follow-up (∼60 %, both groups) might affect the generalizability of the study. However, analyses showed that non-returners of questionnaires at 12 months had significantly lower scores on HSCL depression at baseline compared to returners. Multiple analyses were performed, possibly increasing the risk of finding low *p* values by coincidence. The patients in the MI group received more therapist time, which may have influenced the results. For practical reasons, there was no blinding of patients or therapists for the different treatments. The treatment sessions were unfortunately not videotaped, but therapists in the BI group had been videotaped previously [22]. Manuals were written for both treatments to ensure equal practice of the methods. The BI group had fewer and more experienced therapists compared to the MI group; however, the therapists in the MI group had regular meetings and supervision. The first author developed the ISIVET and treated 29 patients. However, the outcomes were based on the questionnaires that patients filled in at home before the consultations, not on scores in the ISIVET.

## Conclusions

The results indicate that the new MI may represent an important supplement in the multidisciplinary therapeutic work in patients with chronic musculoskeletal pain and that visualization, shared decision and multidisciplinary assessment can reinforce the effect of treatment. The MI with the ISIVET should be applied in new studies to see if results could be reproduced or improved further.
